# Pediatrician level of knowledge on management of type 1 diabetes mellitus and diabetic ketoacidosis

**DOI:** 10.1186/1687-9856-2013-S1-P3

**Published:** 2013-10-03

**Authors:** Ratna Dewi Artati, Indah S Widyahening, Aman B Pulungan

**Affiliations:** 1 Department of Child Health, Medical School, University of Hasanuddin, Makassar, Indonesia; 2 Department of Community Medicine,Medical School,University of Indonesia, Jakarta, Indonesia; 3 Department of Child Health, Medical School, University of Indonesia, Jakarta, Indonesia

## Background

Diabetes Mellitus (DM) is a metabolic disorder characterized by high blood glucose level that result from defects in insulin secretion and/or action. Prevalence of type 1 DM (T1DM) has increased every year, including in Asian countries. Effective management of T1DM needs balancies of insulin treatment, diet, physical activities, and education. Physician that involve in T1DM management should to have adequate competencies and should be able to provide specific attentions as T1DM is a condition that may need long-term monitoring and treatment as well as may have potency of complications, both acute and chronic.

## Objective

To examine pediatrician level of knowledge on T1DM and diabetic ketoacidocis (DKA) management and factors that influence their level of knowledge.

## Methods

Research design was a cross-sectional study. Data collected in Banten and Surabaya from August to September 2009. Study population were pediatricians who attended trainings on management of T1DM and DKA in Banten and Surabaya and pediatricians among them that agreed to fill and return study questionnaire and knowledge data sheet were included as sample of this study. Bivariate analysis with X^2^ or Fisher Exact test (significant value p ≤ 0,05) were analysis methods applied in the study.

## Results

There were 64 subject included in the study, men 31 (48,4%) and woman 33 (51,6%). We found that younger participants have good level of knowledge with OR 3,64 (95% CI 1,29 to 10,26) and subject with shorter length of duty with good level of knowledge with OR 7,42 (95% CI 2,13 to 25,84).

## Conclusion

There is association between younger age and shorter length of duty with pediatrician level of knowledge on T1DM and DKA management.

